# 

**Published:** 1877-05

**Authors:** 


### Contents of May Number.

### ORIGINAL.

ART. I.—The Therapeutics of Periodical Cephalalgia, by F. Bradnack, M. D.,........ 243
ART. II.—Report of a Case of Sub-Luxation of the Inferior Maxilla Backwards, by E. M. Moore, Jr., M. D.,........ 255
ART. III.—Abstract of the Proceedings of the Buffalo Medical Association,........ 258

### MISCELLANEOUS.

The Veneral Ulcer, or Chancroid,........ 262
Shortening of the Lower Limb after Fracture of the Femur,........ 268
Concerning "Blue Glass,"........ 271
Salicylic Acid,........ 272
State Boards of Health,........ 274
Symptoms Resulting from Anæthesia by Ether in Young Subjects,........ 274

### EDITORIAL.

Progress, or Something New,........ 274
American Medical Association,........ 275
One Thousand Dollars Reward,........ 276
BOOKS REVIEWED:
The Transactions of the American Association,........ 277
Healthy Skin,........ 278
The Tonic Treatment of Syphilis,........ 280
Gross on the Urinary Organs,........ 281
Books and Pamphlets Received,........ 282

